# The impact of geographic access on institutional delivery care use in low and middle-income countries: Systematic review and meta-analysis

**DOI:** 10.1371/journal.pone.0203130

**Published:** 2018-08-30

**Authors:** Teketo Kassaw Tegegne, Catherine Chojenta, Deborah Loxton, Roger Smith, Kelemu Tilahun Kibret

**Affiliations:** 1 Department of Public Health, College of Health Sciences, Debre Markos University, Debre Markos, Ethiopia; 2 Research Centre for Generational Health and Ageing, School of Medicine and Public Health, University of Newcastle, Newcastle, New South Wales, Australia; 3 Mothers and Babies Research Centre, School of Medicine and Public Health, University of Newcastle, Newcastle, New South Wales, Australia; 4 Department of Public Health, College of Medical and Health Science, Wollega University, Nekemte, Ethiopia; University of Toronto, CANADA

## Abstract

**Background:**

Geographic access to obstetric care facilities has a significant influence on women’s uptake of institutional delivery care. However, this effect was not consistent across studies. Some studies reported that geographic access to obstetric care facilities had no influence on the use of facility delivery. Therefore, this systematic review and meta-analysis synthesized and pooled the influence of geographic access on institutional delivery service uptake in low and middle-income countries.

**Methods:**

Multiple combinations of search terms were used to search articles from six databases and a hand search of reference lists performed. We included observational studies conducted in low and middle-income countries which reported the influence of geographic access on delivery care use. The pooled effects of geographic access on institutional delivery care use were calculated using a random-effects model with a 95% confidence interval.

**Findings:**

In this study a total of 31 studies were included. Among these studies, 15 met criteria for inclusion in the meta-analyses, while the remaining 16 were summarized using qualitative synthesis. Studies included in the analysis where women had to walk 60 minutes or less to access a health facility delivery were significantly heterogeneous. Having access to obstetric care facilities within five kilometres was significantly associated with institutional deliveries (pooled OR = 2.27; 95% CI = 1.82, 2.82). Similarly, a travelling time of 60 minutes or less was significantly associated with higher odds of health facility delivery (pooled OR = 3.30; 95% CI = 1.97, 5.53). Every one-hour and one-kilometre increase in travel time and distance, respectively, was negatively associated with institutional delivery care use.

**Interpretation:**

Geographic access measured in either physical distance and/or travel time was significantly associated with women’s use of facility delivery. The greater the distance and/or travel time to obstetric care facilities, the greater the barrier and the lesser the service uptake.

## Background

The distribution of healthcare facilities must be based on equity. All geographical areas, economic and ethnic groups should have equal access to healthcare services irrespective of any preconditions [1]. However, healthcare facilities are not evenly distributed globally. The distribution varies significantly in low and middle-income countries [2, 3] where rural areas have the least access to healthcare services [4–7]. For instance, the global met need for emergency obstetric care (EmOC) is 45%; the gap is very high between low (21%) and high-income (99%) countries [6]. Countries with high and moderate numbers of maternal deaths have an insufficient number of Basic Emergency Obstetric and Neonatal Care (BEmONC) facilities and the available EmOC facilities did not provide the full range of signal functions [4].

People living in low and middle-income countries (LMICs) tend to have less access to health care services than those in high-income countries [8]. Even within these countries, the poor and those living in rural areas have less access to services relative to those living in urban areas [8, 9]. In rural areas, and crowded urban centres, the geographical dimension of access could be more important than in urban centres with good transportation infrastructure; in such settings, service users might be expected to walk long distances and/or spend more time travelling [9]. The availability of transport services, the nature of roads (seasonal impassibility), mountains and rivers may also play a role in determining access to health care services [10].

The delays in deciding to seek care, reaching healthcare facilities and getting adequate care at health facilities play an important role in healthcare utilization [11]. Investments to improve access to and quality of health services also play a significant role in improving the health status of a population [12]. Healthcare access is defined in terms of geographic, financial, temporal, digital/eHealth and cultural access, and availability of specific services [8, 9]. Availability is the opportunity to get the right type of healthcare, with appropriate healthcare providers, materials and equipment [8]. The geographic dimension of health service access is the measure of physical distance and/or travel time to service delivery points [8, 9].

For a variety of reasons, the nearest health facility might not be the facility of choice. Not all healthcare services are provided at all health facilities [10]. For instance, in many countries, comprehensive emergency obstetric care (CEmOC) is not available at the lowest level health facilities. In most countries, emergency obstetric care is only available at hospitals, and for this reason pregnant women and their families may be required to travel a long distance for childbirth [10].

Studies from Zambia and Malawi found that the odds of facility delivery were higher among those who had close physical access to higher-level healthcare facilities [13, 14]. Women’s use of facility delivery in Haiti was higher among those who were living within 10 kilometres of a health facility with the highest level of readiness score to provide delivery care [15]. Similarly, in Malawi and Zambia living close to a facility providing delivery services was significantly associated with health facility delivery [14]. It was also noted that an increase in geographic distance was associated with a decrease in the use of health facility delivery [13, 16].

Not all studies, however, have found that closeness is related to service uptake. Community-based cross-sectional studies in different countries have found that having access to obstetric care facilities within one-hour [17, 18] and three kilometres [19, 20] of travel were not significantly associated with institutional delivery. Different studies used different measures of physical access; some used physical distance and others travel time. Even those studies who used a similar measurement (for instance, physical distance) used different cut-off points. Therefore, this study synthesized and pooled the evidence on the influence of geographic access on institutional delivery care use in low and middle-income countries.

## Methods

### Search strategy

The search strategy included the following databases: MEDLINE, EMBASE, CINAHL, PsycINFO, Scopus and Maternity & Infant Care. Multiple combinations of search terms or keywords, such as delivery or obstetric care, childbirth, geographic/physical access or proximity, observational studies, low and middle-income countries, and Boolean operators were used (see S1 Table). The search terms/keywords first used in OVID MEDLINE were adapted to the other databases mentioned above. In addition to this, a hand search of reference lists was carried out.

### Study selection

Search results were imported into EndNote software to aggregate relevant articles and to manage duplications. Two authors independently screened the titles and abstracts to determine if the returned electronic search articles were related to the study. The respective lists of articles of both authors were combined and full-text articles were reviewed against the inclusion and exclusion criteria. Disagreements were resolved through discussion with a third reviewer.

### Inclusion and exclusion criteria

Studies published in English and conducted in LMICs as defined by the World Bank [21] were included. Quantitative cross-sectional studies, cohort and case-control studies published since January 1, 2000 (the year the Millennium Development Goals (MDGs) were introduced) up to December 31, 2016 were included. The article search, for all the above-mentioned databases, was started on May 23, 2017 and ended on September 18, 2017. The most recent articles included in this paper were published in 2016. There was no experimental/ interventional study identified in the search process. Organization reports were excluded in this analysis. To be included, the studies had to report on the influence of geographic accessibility on maternal delivery service use.

### Outcome measures

Articles that reported geographic access on institutional delivery care use were selected. The measurement of the study outcome was utilization of institutional delivery care.

### Assessment of risk of bias

The methodological quality of the included studies was assessed using the Joanna Briggs Institute critical appraisal (**assessment** of **risk** of **bias**) checklists. The Joanna Briggs Institute, which is internationally known as JBI, along with its collaborators developed a systematic review reviewer’s manual. The aims of the JBI critical appraisal tools are to assess the methodological quality of a study and to determine to what extent a study addressed the possibility of bias in its design, conduct and data analysis [22]. For instance, JBI has critical appraisal checklists for prevalence studies, cohort and case-control studies [22]. The critical appraisal checklist for prevalence studies has nine criteria with options of Yes, No, Unclear or Not Applicable for each individual prevalence study. Based on this individual study assessment, an overall appraisal, either to be included or excluded, is given to that particular study [23]. Similarly, the JBI appraisal checklist has 11 criteria for cohort [24] and 10 criteria for case-control [25] studies. Therefore, two authors independently assessed the methodological quality of each study using the JBI critical appraisal checklist for studies reporting prevalence data [23], cohort [24] and case-control [25] studies. Disagreements were resolved by discussion with a third reviewer.

### Data extraction

Data on the influence of geographic accessibility on maternal delivery care use were extracted. A data extraction form that included general information (publication details and country), and specific information (study setting, study design, study population, sample size, main findings) was used (Table 1). A summary matrix with the data extracted from all individual studies was created. Two authors independently extracted the data from the included studies into the constructed matrices. Discrepancies were resolved by discussion and the original study was reviewed to resolve further discrepancies.

**Table 1:** Summary table for studies included in the systematic review and meta-analysis.

Study and country	Study design and setting	Study population and sample size	Results	Geographic access on delivery care use	Remark (quality)
Kawakatsu et al, 2014; Kenya[26]	Community based cross-sectional study	2026 women who had children aged from 12–24 months	48% were institutional delivery	≤20 minute travel time was associated with increased institutional delivery; AOR = 2.48: 1.74–3.55 Ref: >60minutes	**9/9**
Hailu & Berhe, 2014; Ethiopia[27]	Community based cross-sectional study	485 reproductive age women who had birth two years preceding the survey	31.5% gave birth at health facilities	An increased institutional delivery for <60 minute of travel to nearest health facility; AOR = 3.3: 1.15–9.52 Ref: ≥60minutes	**8/9**
Habte & Demissie, 2015; Ethiopia[28]	Community based cross-sectional study	816 women who had birth two years preceding the survey	31% of births were in health facility	Decreased institutional delivery for >60 minute travel time; >60 minute; AOR = 0.22: 0.09, 0.55 Ref: <30 minute	**7/9**
Joshi et al, 2016; Nepal[29]	Community based cross-sectional study	275 women who had birth five years preceding the survey	35% had delivered at health facility	Increased institutional delivery for ≤60 minutes of travel time to nearest delivery health facility; AOR = 7.7: 4.1, 14.4 Ref: >60 minute	**3/9**
Wagle et al, 2004; Nepal[30]	Community based cross-sectional study	308 women who had birth within 45 days of the survey	50.6% of deliveries were in health facility	A traveling time of >60 minutes to a maternity hospital led to an increased odds of home delivery; AOR = 7.9; 3.7, 16.6 Ref: <60 minutes	**7/9**
Jain et al, 2015; Pakistan[31]	Cross-sectional study, Linked health facility & household survey	763 obstetric care facilities and 4435 women who had birth three years before the survey	21.0% of women had no access to delivery care facility within 10 kilometre	Odds of institutional delivery decreased by 3% with one kilometre increase in distance; AOR = 0.97 Having access to basic (AOR = 1.79) or comprehensive (AOR = 1.72) emergency obstetric care within 10 kilometre increased odds of institutional deliveries	**9/9**
De Allegri et al, 2011; Burkina Faso[32]	Community based cross-sectional study	435 women reported pregnant 12 months prior to the survey	72% were health facility delivery	Having access to obstetric care facility within 5km was associated with increased institutional delivery; AOR = 28.42, Robust Standard Error = 11.90	**4/9**
Lohela et al, 2012; Malawi & Zambia[14]	Cross-sectional study, Linked health facility & household survey	Firstborn for multiple births: 8537 deliveries in Malawi, 3682 deliveries in Zambia	52.1% in Malawi & 32.5% in Zambia were health facility delivery	Health facility delivery decreased by 65% for every 10 km increase in distance; AOR = 0.35 (**Malawi)** Health facility delivery decreased by 27% for every 10 km increase in distance; AOR = 0.73 (**Zambia)**	**9/9**
Gabrysch et al, 2011; Zambia[13]	Cross-sectional study, linked analysis (HHS & SPA)	3682 births (firstborn were included in case of multiple births) 1131 health facilities	32.5% births were health facility	Every doubling in travel distance was associated with a 29% decrease in institutional delivery	**9/9**
Anyait et al, 2012; Uganda[19]	Community based cross-section study	500 women who had birth two years preceding the survey	45.4% delivered in health facility	Having access to obstetric care facility within 3km **is not associated with facility delivery** Crude OR = 1.9: 1.2, 3.1	**7/9**
Joharifard et al, 2012; Rwanda[33]	Community based cross-sectional study–Trend analysis	3106 lifetime deliveries from 895 women (18–50 years of age and gave birth within three years)	89.8% of them delivered in health facility	Facility delivery decreased per a single kilometre increase in distance to the closest health facility; AOR = 0.909 (0.846, 0.976)	**5/9**
Zegeye et al, 2014; Ethiopia[16]	Community based cross-sectional study	528 women who gave birth preceding the survey	8% of mothers gave birth in health facility	A 22% decrease in institutional delivery per one kilometre increase in walking distance to the nearest health centre; AOR = 0.78 (0.64, 0.96)	**7/9**
Masters et al, 2013; Ghana[34]	Cross-sectional study; Linked population and health facility data	1172 mothers, and 1646 births, and 1268 facilities	39.0% were in facility deliveries	An increase in travel time of one hour decreased the odds of facility delivery by 20%; AOR = 0.80: 0.69, 0.93	**9/9**
De Allegri et al, 2015; Burkina Faso[35]	Community based cross-sectional study	420 women of recent history of childbirth	11% of home delivery	A distance of ≥7 km was significantly associated with an increased in home delivery; AOR = 19.33; 3.37, 110.88	**6/9**
Worku & Alemay, 2016; Ethiopia[36]	Community based cross-sectional study	573 women who had birth one year preceding the survey	16.9% were health facility births	Travel time to closest health facility: Ref; >2 hour <60 minutes: AOR = 5.2; 2.8, 12.3	**6/9**
Van et al, 2006; Kenya[37]	Community based cross-sectional study	635 women who had birth one year preceding the survey	83% were outside health facility	Travel time: Birth outside health facility Ref: <60 minutes of walk >60 minutes of walk: AOR = 2.75; 1.33, 5.68	**5/9**
Lwelamira et al, 2012; Tanzania[38]	Community based cross-sectional study	984 women gave birth 2 years prior	54% were in institutional deliveries	Access beyond 10 km; OR = 0.62: 0.47, 0.81 Ref: <5km	**7/9**
Yanagisawa et al, 2006; Cambodia[39]	Community based cross-sectional study	980 women aged 15–49 who gave birth within 3 months	55.2% were health facility	Distance to Health Centre is for facility delivery; Ref: >5km <2km; OR = 3.35; 2.10, 5.34 Distance to Hospital is for facility delivery; Ref: ≥20km: <10km: OR = 3.32: 2.02, 5.45	**8/9**
Gage & Guirle, 2006; Haiti[40]	Community based cross-sectional study	4533 rural women aged 15–49 years	9.6% were intuitional deliveries	Distance to hospital; Ref <5km 5–14 km: OR = 0.339; 0.197, 0.584	**9/9**
Kesterton et al, 2012; India[41]	Community based cross-sectional study	98777 & 90303 reproductive age women who had births within 3 years of survey	Trend (1989 to 1998) is 15–25%	Distance to hospital: Ref: >30km ≤5km: OR = 2.43; 1.93, 3.06	**9/9**
Mageda & Mmbaga, 2015; Tanzania[42]	Community based cross-sectional study	598 women who had birth one year preceding the survey	56% were health facility births	Distance to health facility: Ref; ≥10km <5km; OR = 2.3: 1.3, 3.9	**8/9**
Faye et al, 2011; Senegal[43]	Community based cross-sectional study	373 women who had childbirth in the last 12months	22% were home delivery	Distance to health centre; Home births >5km; OR = 2.24; 1.21, 4.15 Ref: ≤5km	**6/9**
Kitui et a, 2013; Kenya[20]	Community based cross-sectional study	3967 reproductive age women who had births within 5 years preceding the survey	46.8% were health facility births	Distance to health facility: AOR not significant 2-5km: COR: 0.5: 0.46, 0.68 Ref: <2km	**9/9**
Ogolla, 2015; Kenya[44]	Community based cross-sectional study	600 women aged 15–49 who had births within 6 months prior	33.3% were health facility births	Distance to nearest health facility; Ref: ≤10km >10 km; OR = 0.5: 0.3, 0.7	**9/9**
Kumar et al, 2014; India[45]	Community based cross-sectional study	158897 women aged 15–49 years	36% were institutional births	A one kilometre increase in distance is associated with a 4.4% decrease in health facility delivery	**9/9**
Hounton et al, 2008; Burkina Faso[46]	Community & health facility based cross-sectional study	43 Health Facilities & census of women aged 12–49	81536 births; 3145 (38.4%) were institutional births	Institutional birth decrease with… Odds ratio/km; distance to health centre 0.77/km (<7.5km) & 0.97/km (≥7.5km) Distance to hospital; Odds ratio/10km = 0.83	**9/9**
Teferra et al, 2012; Ethiopia[17]	Community based cross-sectional study	371 women who had birth one year preceding the survey	12.1% were health facility births	Travel time to closest health facility: AOR not significant <60 minutes: COR = 6.2; 1.87, 20.5 Ref: ≥60 minute	**9/9**
Amano et al, 2012; Ethiopia[18]	Community based cross-sectional study	855 women who had birth one year preceding the survey	12.3% were health facility births	Travel time to closest health facility: AOR not significant ≤30 minutes: COR = 2.04; 1.26, 3.30 Ref: >30 minute	**8/9**
Shimazaki et al, 2013; Philippines[47]	Community based cross-sectional study	354 women who had birth in the 3 years	44.4% were HF delivery	Time taken to nearest HF; Ref: ≥31minutes 11–30 minutes; OR = 3.3; 1.7, 6.6 ≤10minutes: OR = 6.9; 3.4, 14.2	**6/9**
Karkee et al, 2013; Nepal[48]	Community based prospective cohort study	644 pregnant women up to 45 days of postpartum period	547 (85%) of them gave birth at health facility	≤30 minute travel time was significantly associated with health facility delivery; AOR = 11.61: 5.77–24.04 Ref: >60minutes Inverse of ≤30 (take Ref: ≤30 minute) >60minutes: AOR = 0.09: 0.04, 0.17	**4/11**
Feyissa & Genemo, 2014; Ethiopia [49]	Unmatched case control	320 women aged 15–49 years	80 cases (institutional) and 240 home deliveries	≥10 km; OR = 0.665: 0.173, 0.954 Ref: <5km Travel time: Ref; >2hour <60 minute; AOR = 3.554; 0.884, 14.283	**7/10**

### Data analysis

The results of studies were extracted, reviewed and reported in a systematic format. A Preferred Reporting Items for Systematic Reviews and Meta-Analysis (PRISMA) checklist [50] was used to synthesize and report the findings. This analysis was aimed to give a qualitative and quantitative synthesis. The qualitative synthesis was done for the 16 articles which were not included in the meta-analysis procedure. The results of this synthesis, along with studies included in the meta-analysis procedure, are presented in an evidence table (Table 1) and narrated in detail. A meta-analytic procedure was used to compute and aggregate effect sizes. The pooled effect size (Odds Ratio—OR) was calculated using a random effects model. The adjusted odds ratio estimates of each individual study were used in this meta-analysis. The Q statistics, I^2^ and Tau squared (τ^2^) were used to examine the heterogeneity of studies. The analysis was done using ProMeta software, version 3.0.

## Results

Three hundred and ninety-three articles were retrieved, from which 33 duplicates were removed. Three hundred and ten articles were excluded based on title and abstract. The remaining 50 articles were reviewed using the full text. Nineteen articles were excluded; amongst these studies, four were descriptive studies, six studies did not report geographic access, six were not for the general population, and three studies did not define their outcome variable clearly. For instance, four were focused only on skilled birth attendance [51–54], and two were on unintended pregnancies [55] and mother to child transmission of HIV [56].

**Fig 1:**
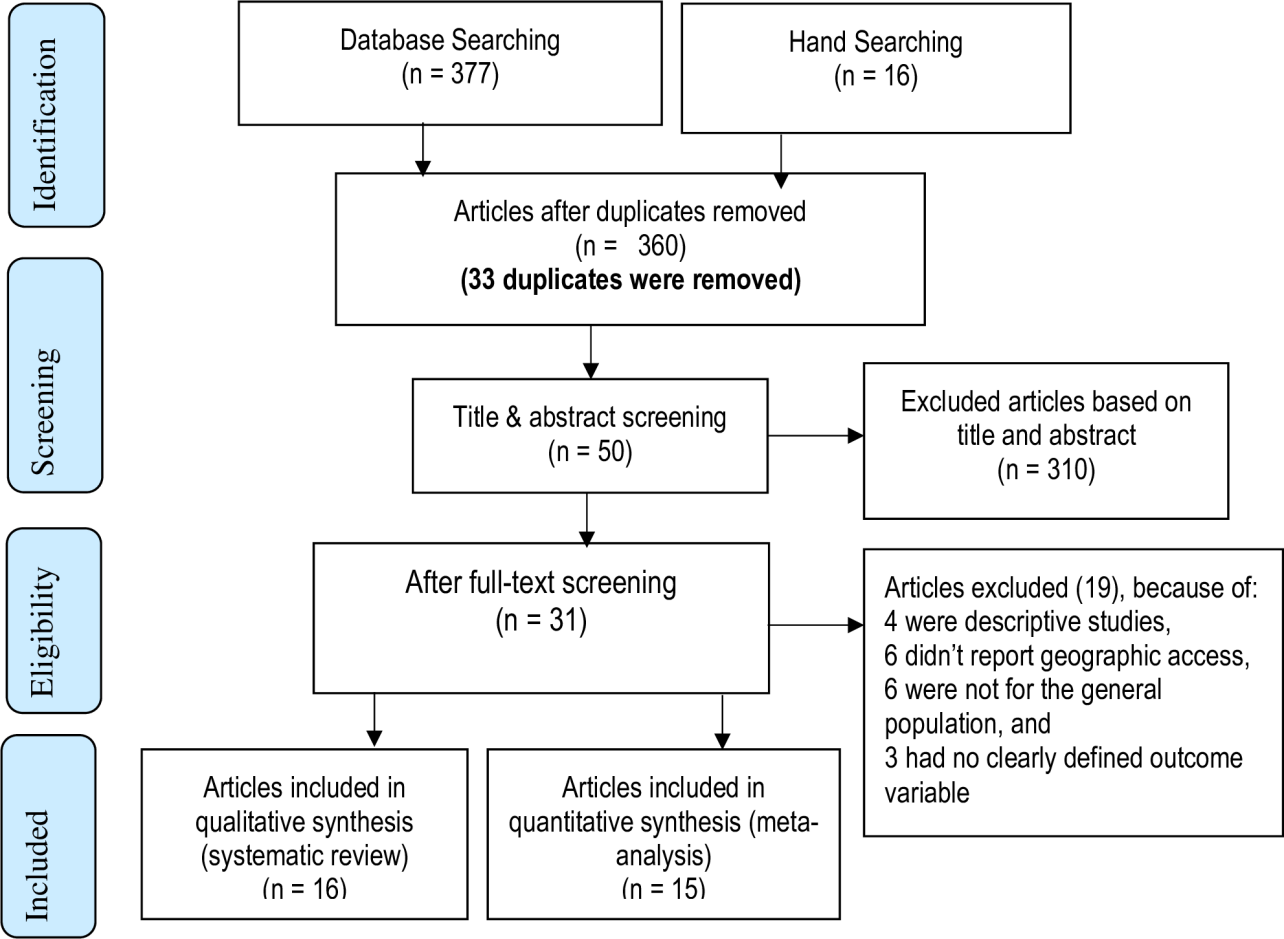
Systematic review and meta-analysis flow diagram adapted from the 2009 PRISMA statement (42).

Thirty-one studies were identified which fulfilled the eligibility criteria; 16 were included in the qualitative synthesis (systematic review) and 15 in the quantitative synthesis (meta-analysis) (Fig 1). Data extracted from the 31 studies are shown in Table 1. Four of the 31 studies were a linked analysis of population and health facility surveys [13, 14, 31, 34] (Table 1). With the exception of three studies [17–19], all showed a significant association between physical access and delivery care use.

In this analysis, all the 31 included articles were conducted from 2004 to 2016. Amongst these, 26 were conducted from 2011 to 2016. With regard to the study design, 24 of the included studies were community-based cross-sectional studies whereas five studies were a linked analysis of community-based and health facility data (Table 1).

All the studies included in both the systematic review and meta-analysis measured the association of geographic access on institutional delivery care use. The main difference was the measurement of the exposure variable (geographic access—distance/travel time). For example, eight studies included in the systematic review treated the independent variable (geographic access) as a continuous variable. However, the 15 studies included in the meta-analysis used cut-off points, 60 minutes of walk and 5km distance, to measure the impact of time and distance on institutional delivery care use (Table 1).

### Assessment of risk of bias

The methodological quality of the included studies was evaluated using the JBI critical appraisal checklist for cross-sectional, cohort and case-control studies [23–25], which resulted in an average score of 63.04%. Of the included studies, only three were graded as poor quality [29, 32, 48]. The risk of bias or quality assessment grading for the different components of each study is shown in S2 Table, S3 Table and S4 Table.

### Impact of geographic access on delivery care use

#### Having access to an obstetric care facility within 60 minutes’ walk

**Fig 2:**
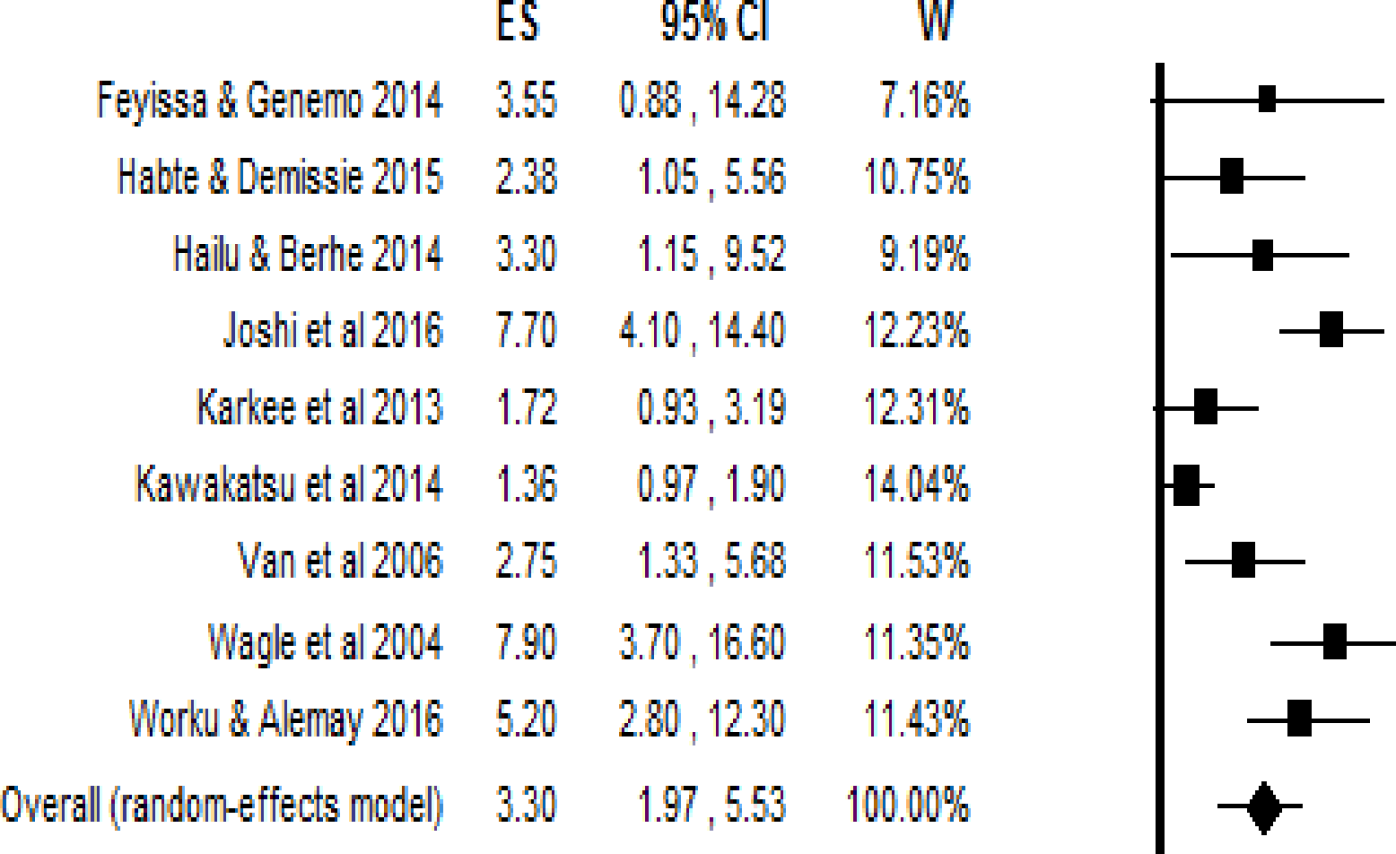
The effect of one hour and less travel time on delivery care use, effect sizes with 95% confidence interval.

**Fig 3:**
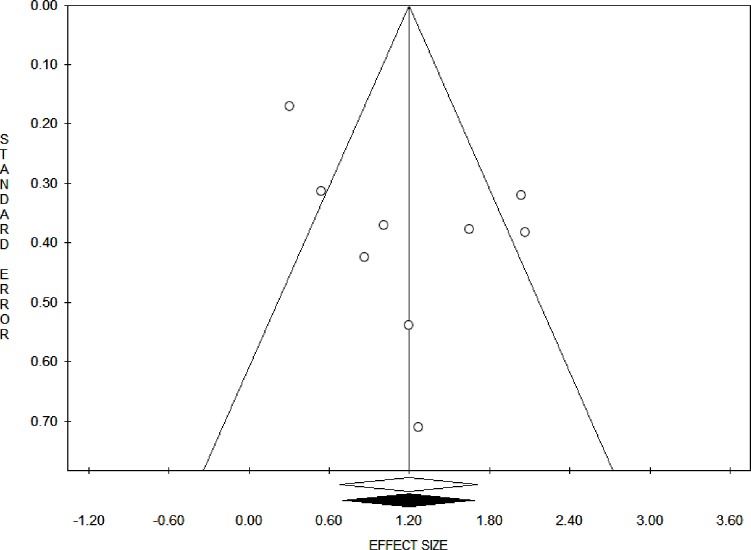
Funnel plot on having access to obstetric care facility within one hour’s travel time.

The pooled estimates (Odds Ratio) showed that the impact of geographic access on institutional delivery care use was 3.30 (95% Confidence interval = 1.97, 5.53). This indicates that pregnant women who had access to obstetric care facilities within a 60-minute walk had 3.3 times the odds of giving birth at health institutions (Fig 2). The Trim and Fill analysis found that there is no need for additional studies to balance the symmetry of the funnel plot. Both the Funnel plot (Fig 3) and the Egger’s test showed that there is no publication bias in the included studies (P-value = 0.08).

Studies included estimating the pooled effect of access to obstetric care facility within 60 minutes of travel showed that they were statistically heterogeneous. The Q-value was 39.69 with 8 degrees of freedom and P-value = 0.000. The I^2^ statistic (a measure of the proportion of the variance in the observed effects that is due to the variance in the true effects), was 79.84, which demonstrates that about 80% of the variance in the observed effects was due to the variance in the true effects. Tau squared (τ^2^) is the variance of the true effect sizes, whereas Tau (τ) is the standard deviation of the true effects (both in log units). The estimated τ^2^ and τ were 0.47 and 0.68, respectively. The prediction interval was from 0.58 to 18.74. Therefore, in most populations, we would expect that the odds ratio for delivery care use would fall from 0.58 to 18.74.

#### Having access to an obstetric care facility within 5 kilometers

**Fig 4:**
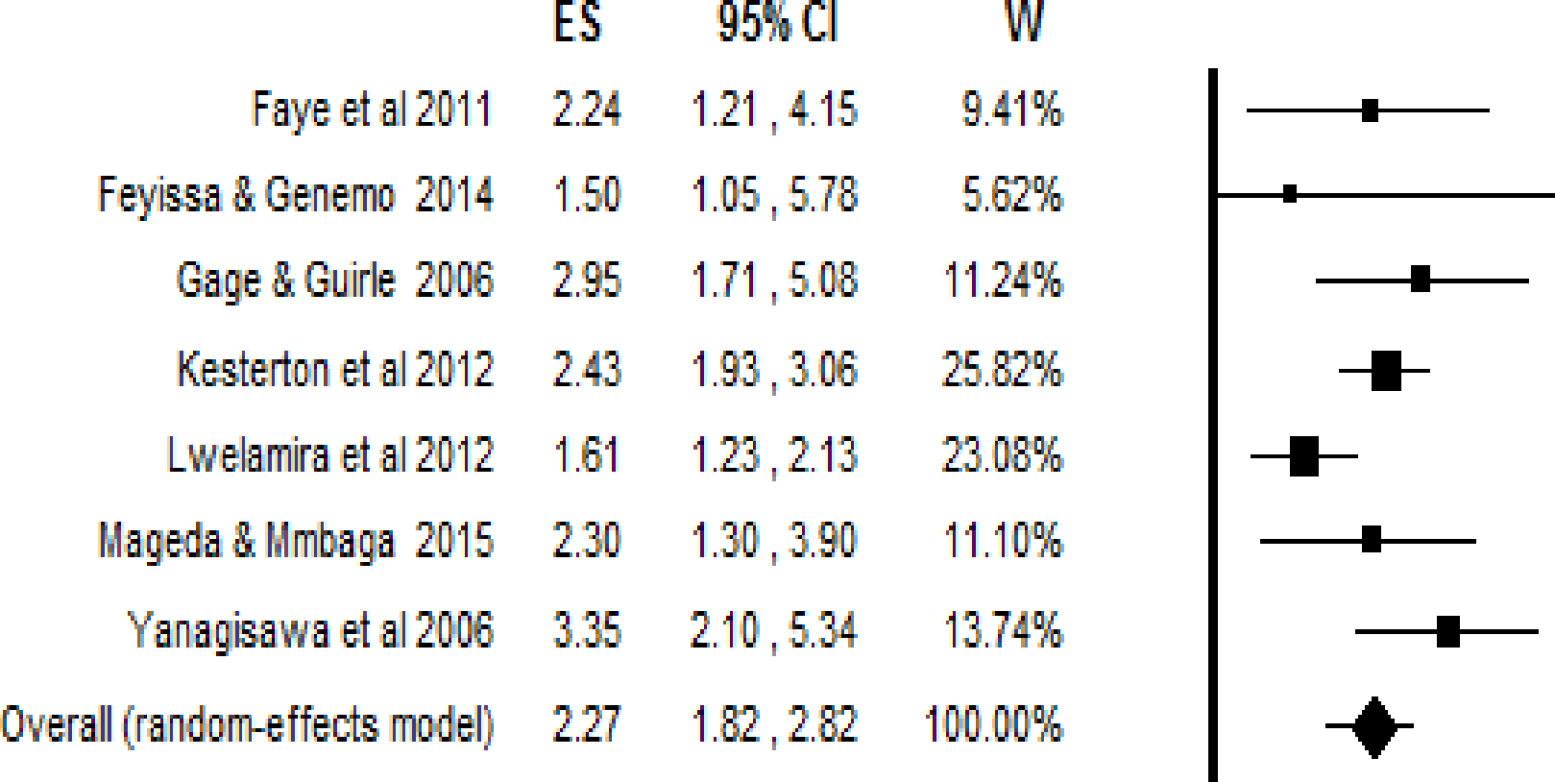
The effect of geographic access within 5km on delivery care use, effect sizes with 95% confidence interval.

**Fig 5:**
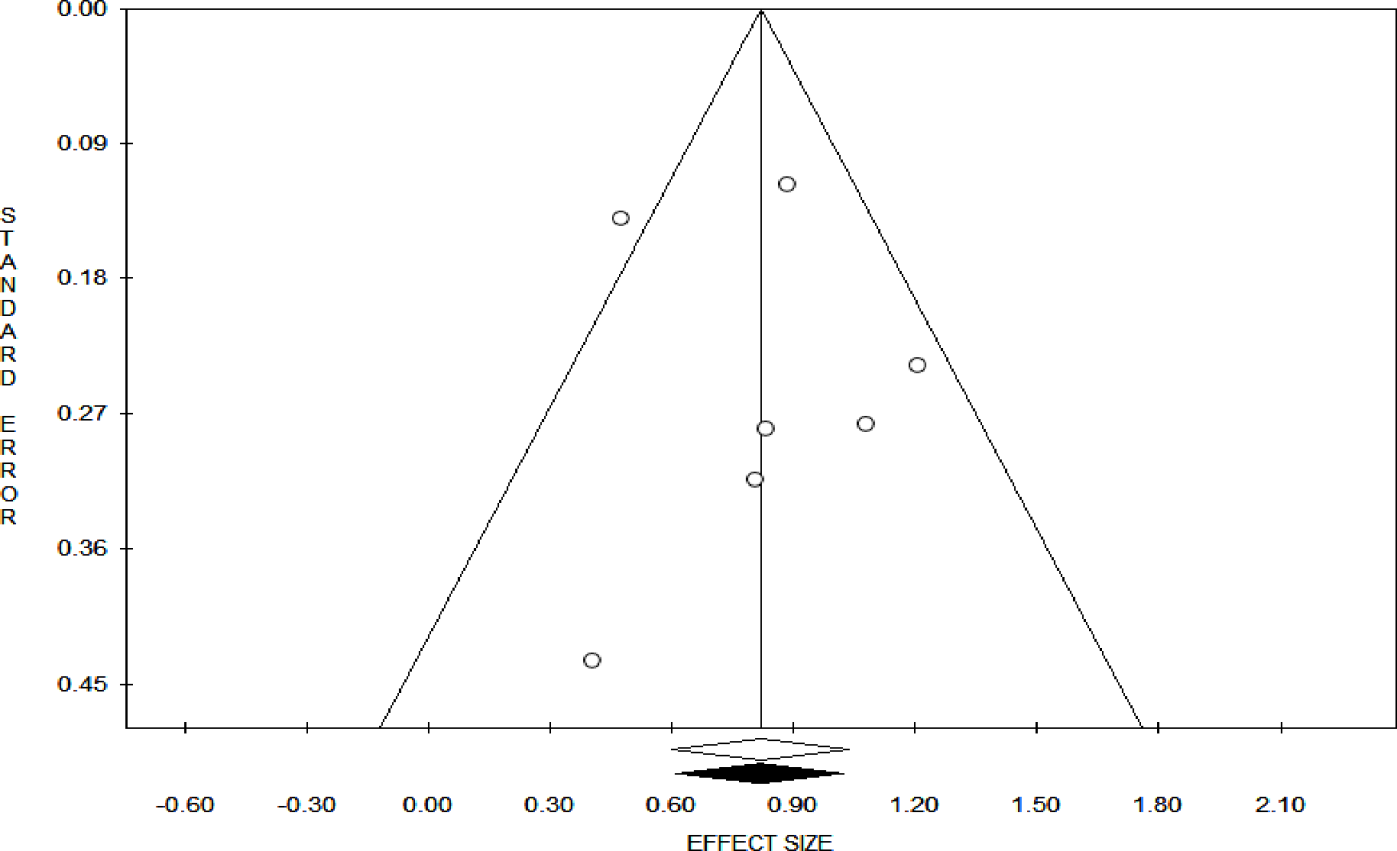
Funnel plot on geographic access to obstetric care facility within 5km.

The pooled estimate found that pregnant women who had access to obstetric care facilities within 5km had 2.3 times the odds of giving birth at healthcare facilities (95% Confidence interval = 2.27; 1.82, 2.82) (Fig 4). The Trim and Fill analysis found that no more studies are required to balance the symmetry. Both the Funnel plot (Fig 5) and the Egger’s test showed that there is no publication bias in the included studies (P-value = 0.74).

In case of women’s access to an obstetric health facility within five kilometers of their usual place of residence, the included studies were not statistically heterogeneous. The estimated Q-value was 10.71 with 6 degrees of freedom and P-value = 0.098. The estimated I^2^ was 43.99, which shows that about 44% of the variance in the observed effects was due to the variance in the true effects. The estimated τ^2^ and τ were 0.03 and 0.19, respectively. The prediction interval was from 1.34 to 3.85. Therefore, in most of the population, we would expect that the odds ratio for delivery care use would fall between 1.34 and 3.85.

#### Qualitative synthesis

Furthermore, the qualitative synthesis of most of the studies showed that geographic access had an effect on obstetric care use. Physical access to obstetric care facilities was assessed in two ways: in terms of geographic distance and travel time to health care facilities. When the travel time falls to half an hour or less, pregnant women were more likely to have a health facility delivery [47]. However, in another two studies, having access to a delivery care facility within one-hour [17, 18] was not associated with institutional delivery. Every one-hour increase in travel time to the nearby obstetric care facilities was associated with a 20% decrease in the odds of facility delivery [34].

In addition to travel time, a one-kilometre increase in walking distance to obstetric care facilities was significantly associated with a decrease in health facility delivery [16, 31, 33, 45, 46]. In rural Zambia, every doubling of distance to the nearest obstetric care facility was significantly associated with a 29% decrease in health facility delivery [13]. Moreover, the odds of health facility delivery decreased by 65% in Malawi and 27% in Zambia for every ten kilometre increase in distance to the closest obstetric care facility [14].

The odds of a health facility delivery were higher among pregnant women who had physical access to obstetric care facilities within ten [31, 44] and five [32] kilometers. However, having access to obstetric care facilities within three [19] and two kilometers [20] was not significantly associated with institutional delivery care use. In Burkina Faso, pregnant women who resided seven or more kilometers away from obstetric care facilities were more likely to have home births than those living further away [35].

## Discussion

The main findings of this meta-analysis and systematic review were that geographic access to obstetric care facilities, measured in either physical distance and/or travel time, had an impact on institutional delivery use. This study was the first of its kind to synthesize and pool the influence of geographic access on institutional delivery care uptake measured using two different methods. The influence of geographic access on institutional delivery care uptake was pooled using two different measurement cut-off points. A five-kilometre distance and a one-hour travel time were used to make the comparison clearer and more uniform across different studies.

Pregnant women who were living within five-kilometers of an obstetric care facility had higher odds of institutional delivery as compared to those living beyond a physical distance of five-kilometers. In terms of walking time, the odds of using institutional delivery were high among pregnant women who had access to obstetric care facilities within an hour’s walk. This implies that long distance has a dual influence on institutional delivery service utilization. It can be a barrier for both reaching obstetric care facilities and discouraging seeking care. The problem worsens for rural pregnant women, who often have no access to reliable transportation systems [9]. Furthermore, it was observed that both a single kilometre and a one-hour increase in accessing obstetric care facilities were associated with lower odds of institutional delivery. This was consistent with the concept of distance decay [57], where service uptakes varied inversely with distance. Therefore, the further away a pregnant woman lives from an obstetric care facility, the less likely she will be to use an institutional delivery service.

Geographic access was a problem in most settings; however, some studies indicated that this was not the case in some settings for the uptake of obstetric care services. For instance, a recent study done in the United Republic of Tanzania found that women living in the more remote areas had increased uptake of institutional delivery between 2007 and 2013 [58]. This indicated that in addition to national health policies to improve maternity care access, other drivers of service uptake such as improvements in the road network [10] and health facilities readiness to provide obstetric care services [15] should also be emphasized. Women’s education, awareness and perceptions of maternal health services [59], socioeconomic status, and media access in the community [60] influence institutional delivery care utilization.

## Limitations of the study

Even though this study is the first of its kind, it has several limitations. It only examined one aspect of the three delays model–delay in reaching health care facilities [11] and measures of healthcare access [8, 9]; furthermore, it did not address the different means of transportation, travel costs and terrains. There were also variations in the operationalization and measurement of geographic access to obstetric care facilities. This was due to the unavailability of a universally agreed clear cut-off point, in either a geographic distance and/or travel time, for a health facility to be considered as accessible or not. The World Health Organization uses distance and travel time as a measure of physical accessibility [61]; however, there was no clear cut-off point for its measurement. Different countries use different cut-off points, for instance, Ethiopia uses a 10 km [62] distance whereas the United States of America and the United Kingdom use 30 minutes travel time [63] for measuring geographic access to health care services.

The analysis was done using both distance and travel time; however, it was still subject to measurement errors. The physical distance used in the studies was not uniformly measured; whilst some studies used a direct physical distance, others used walking tracks. Making comparisons and judgements based on measured physical distance is subject to errors where the geography and transportation infrastructure vary considerably within and among countries. Moreover, though WHO recommends using travel time, instead of physical distance, for assessing geographic accessibility [61] unless variations between and within countries, population groups, and socio-economic factors are considered, there could be problems in making a comparison. The value of, and actual travelling time varies depending on people, for instance, the age and physical condition of the person, and the transportation mode used, and thus its strength as an access barrier also varies.

## Conclusion

This meta-analysis identified that the closer in geographic proximity the obstetric care facilities were located, the more likely that pregnant women were to use institutional deliveries. Future research should account for the different measures of geographic accessibility, taking into consideration means of transportation, travel cost and terrains, for measuring how obstetric care accessibility and utilization of institutional deliveries interact with each other. Further research is needed to compare each of the measures of health care access and how they could influence utilization of obstetric care services. Furthermore, the possible links between geographic access, and quality of obstetric care services, health facilities readiness to provide obstetric care services, and referral systems to a higher level of care in the uptake of obstetric care services should be investigated.

## Supporting information

S1 Table Article search strategy. (DOCX)

S2 Table JBI critical appraisal checklist for studies reporting prevalence Data. (DOCX)

S3 Table JBI critical appraisal checklist for cohort studies. (DOCX)

S4 Table JBI critical appraisal checklist for case control studies. (DOCX)

S1 File Completed PRISMA 2009 checklist. (DOC)
