# Supplementary material for: The impact of geographic access on institutional delivery care use in low and middle-income countries: Systematic review and meta-analysis
Source: PLoS One. 2018 Aug 30;13(8):e0203130. doi: 10.1371/journal.pone.0203130 (PMC6117044; doi:10.1371/journal.pone.0203130)
Supplement: S1 Table — (DOCX) [file pone.0203130.s001.docx]

| **Databases** | **Search Terms** |
| --- | --- |
| **Medline** | Midwifery/ or Maternal Health Services/ or Perinatal Care/ or Delivery, Obstetric/ or Obstetric Labor Complications/ or Labor, Obstetric/ or Caesarean Section/ or Obstetrics/ or Parturition/ or childbirth.mp. or Obstetric care.mp. or delivery care.mp. or delivery assistant*.mp. or birth assistant*.mp. or ((location or place) adj3 (birth or delivery)).mp. **AND** ((geographic or physical) adj5 (location* or access* or proximit* or distance*)).mp. or ((time or distance*) adj5 (walk* or driv* or travel*)).mp. or Health Services Accessibility/ or Spatial access*.mp. or "Catchment Area (Health)"/ or access to care.mp. **AND** Observational Study/ or exp Cohort Studies/ or Case-Control Studies/ or Cross-Sectional Studies/ or ecological stud*.mp. or Randomi*ed trial.mp or Clinical Trial/ or Randomized controlled trial.mp **AND** ((low or middle) adj3 (countr* or nation* or econom*)).mp. OR (Afghanistan or Benin or Burkina Faso or Burundi or Central African Republic or Chad or Comoros or Congo Democratic Republic or Eritrea or Ethiopia or Gambia or Guinea or Guinea-Bissau or Haiti or Korea Democratic People's Republic or Liberia or Madagascar or Malawi or Mali or Mozambique or Nepal or Niger or Rwanda or Senegal or Sierra Leone or Somalia or South Sudan or Tanzania or Togo or Uganda or Zimbabwe or Armenia or Bangladesh or Bhutan or Bolivia or Cabo Verde or Cambodia or Cameroon or Congo Republic or Ivory Coast or Djibouti or Egypt or El Salvador or Ghana or Guatemala or Honduras or India or Indonesia or Kenya or Kiribati or Kosovo or Kyrgyz Republic or Lao PDR or Lesotho or Mauritania or Micronesia Federation or Moldova or Mongolia or Morocco or Myanmar or Nicaragua or Nigeria or Pakistan or Papua New Guinea or Philippines or Samoa or Sao Tome) and Principe) or Solomon Islands or Sri Lanka or Sudan or Swaziland or Syrian Arab Republic or Tajikistan or Timor-Leste or Tonga or Tunisia or Ukraine or Uzbekistan or Vanuatu or Vietnam or West Bank) and Gaza) or Yemen Republic or Zambia or Albania or Algeria or American Samoa or Angola or Argentina or Azerbaijan or Belarus or Belize or Bosnia) and Herzegovina) or Botswana or Brazil or Bulgaria or China or Colombia or Costa Rica or Cuba or Dominica or Dominican Republic or Equatorial Guinea or Ecuador or Fiji or Gabon or Georgia or Grenada or Guyana or Iran or Iraq or Jamaica or Jordan or Kazakhstan or Lebanon or Libya or Macedonia or Malaysia or Malaysia or Maldives or Marshall Islands or Mauritius or Mexico or Montenegro or Namibia or Palau or Panama or Paraguay or Peru or Romania or Russian Federation or Serbia or South Africa or Saint Lucia or Saint Vincent) and the Grenadines) or Suriname or Thailand or Turkey or Turkmenistan or Tuvalu or Venezuela).mp |
| **Embase** | Midwifery.mp. OR midwife/ OR Maternal Health Services.mp.OR health service/ OR perinatal care/ OR obstetric delivery/ OR labor complication/ OR labor/ OR cesarean section/ OR obstetrics/ OR Parturition.mp. OR birth/ or childbirth/ OR obstetric procedure/ OR maternal care/ OR delivery/ OR Delivery care.mp. OR Delivery assistant*.mp. OR Birth assistant*.mp. OR ((location or place) adj3 (birth or delivery)).mp. OR Delivery care.mp. **AND** ((geographic or physical) adj5 (location* or access* or proximit* or distance*)).mp. OR ((time or distance*) adj5 (walk* or driv* or travel*)).mp. ORHealth Services Accessibility.mp. OR health care access/ OR Spatial access.mp. OR Access to care.mp. **AND** case control study/ or observational study/ or cohort analysis/ OR cross-sectional study/ OR Ecological stud*.mp. or Randomi*ed trial.mp or Clinical Trial/ or Randomized controlled trial.mp **AND** ((low or middle) adj3 (countr* or nation* or econom*)).mp. OR (Afghanistan or Benin or Burkina Faso or Burundi or Central African Republic or Chad or Comoros or Congo Democratic Republic or Eritrea or Ethiopia or Gambia or Guinea or Guinea-Bissau or Haiti or Korea Democratic People's Republic or Liberia or Madagascar or Malawi or Mali or Mozambique or Nepal or Niger or Rwanda or Senegal or Sierra Leone or Somalia or South Sudan or Tanzania or Togo or Uganda or Zimbabwe or Armenia or Bangladesh or Bhutan or Bolivia or Cabo Verde or Cambodia or Cameroon or Congo Republic or Ivory Coast or Djibouti or Egypt or El Salvador or Ghana or Guatemala or Honduras or India or Indonesia or Kenya or Kiribati or Kosovo or Kyrgyz Republic or Laos or Lesotho or Mauritania or Micronesia Federation or Moldova or Mongolia or Morocco or Myanmar or Nicaragua or Nigeria or Pakistan or Papua New Guinea or Philippines or Samoa or Sao Tome) and Principe) or Solomon Islands or Sri Lanka or Sudan or Swaziland or Syrian Arab Republic or Tajikistan or Timor-Leste or Tonga or Tunisia or Ukraine or Uzbekistan or Vanuatu or Vietnam or West Bank) and Gaza) or Yemen Republic or Zambia or Albania or Algeria or American Samoa or Angola or Argentina or Azerbaijan or Belarus or Belize or Bosnia) and Herzegovina) or Botswana or Brazil or Bulgaria or China or Colombia or Costa Rica or Cuba or Dominica or Dominican Republic or Equatorial Guinea or Ecuador or Fiji or Gabon or Georgia or Grenada or Guyana or Iran or Iraq or Jamaica or Jordan or Kazakhstan or Lebanon or Libya or Macedonia or Malaysia or Malaysia or Maldives or Marshall Islands or Mauritius or Mexico or Montenegro or Namibia or Palau or Panama or Paraguay or Peru or Romania or Russian Federation or Serbia or South Africa or Saint Lucia or Saint Vincent) and the Grenadines) or Suriname or Thailand or Turkey or Turkmenistan or Tuvalu or Venezuela).mp |
| **PsycINFO** | exp MIDWIFERY/ OR exp Birth/ or Maternal Health Service*.mp. OR Perinatal Care.mp. OR exp Obstetrical Complications/ or exp Obstetrics/ or exp "Labor (Childbirth)"/ or exp Caesarean Birth/ or Delivery, Obstetric.mp. OR exp Obstetrical Complications/ OR Parturition.mp. OR Obstetric care.mp. OR Delivery care.mp. OR Delivery assistant*.mp. OR Birth assistant*.mp. OR ((location or place) adj3 (birth or delivery)).mp. **AND** ((geographic or physical) adj5 (location* or access* or proximit* or distance*)).mp. OR ((time or distance*) adj5 (walk* or driv* or travel*)).mp. OR Health Services Accessibility.mp. OR Spatial access*.mp. OR Access to care.mp. **AND** Observational Study.mp. OR Cohort Study.mp. OR Case-Control Studies.mp. OR Cross-Sectional Studies.mp. OR Ecological studies.mp. or Randomi*ed trial.mp or Clinical Trial/ or Randomized controlled trial.mp **AND** ((low or middle) adj3 (countr* or nation* or econom*)).mp. OR (Afghanistan or Benin or Burkina Faso or Burundi or Central African Republic or Chad or Comoros or Congo Democratic Republic or Eritrea or Ethiopia or Gambia or Guinea or Guinea-Bissau or Haiti or Korea Democratic People's Republic or Liberia or Madagascar or Malawi or Mali or Mozambique or Nepal or Niger or Rwanda or Senegal or Sierra Leone or Somalia or South Sudan or Tanzania or Togo or Uganda or Zimbabwe or Armenia or Bangladesh or Bhutan or Bolivia or Cabo Verde or Cambodia or Cameroon or Congo Republic or Ivory Coast or Djibouti or Egypt or El Salvador or Ghana or Guatemala or Honduras or India or Indonesia or Kenya or Kiribati or Kosovo or Kyrgyz Republic or Laos or Lesotho or Mauritania or Micronesia Federation or Moldova or Mongolia or Morocco or Myanmar or Nicaragua or Nigeria or Pakistan or Papua New Guinea or Philippines or Samoa or Sao Tome) and Principe) or Solomon Islands or Sri Lanka or Sudan or Swaziland or Syrian Arab Republic or Tajikistan or Timor-Leste or Tonga or Tunisia or Ukraine or Uzbekistan or Vanuatu or Vietnam or West Bank) and Gaza) or Yemen Republic or Zambia or Albania or Algeria or American Samoa or Angola or Argentina or Azerbaijan or Belarus or Belize or Bosnia) and Herzegovina) or Botswana or Brazil or Bulgaria or China or Colombia or Costa Rica or Cuba or Dominica or Dominican Republic or Equatorial Guinea or Ecuador or Fiji or Gabon or Georgia or Grenada or Guyana or Iran or Iraq or Jamaica or Jordan or Kazakhstan or Lebanon or Libya or Macedonia or Malaysia or Malaysia or Maldives or Marshall Islands or Mauritius or Mexico or Montenegro or Namibia or Palau or Panama or Paraguay or Peru or Romania or Russian Federation or Serbia or South Africa or Saint Lucia or Saint Vincent) and the Grenadines) or Suriname or Thailand or Turkey or Turkmenistan or Tuvalu or Venezuela).mp |
| **Maternity & Infant Care** | Midwifery.mp. OR Maternal Health Services.mp. OR Perinatal Care.mp. OR Delivery, Obstetric.mp. OR Labor, Obstetric.mp. OR Caesarean Section.mp. OR Obstetrics.mp. OR (Parturition or childbirth).mp. OR (Obstetric care or delivery care).mp. OR (Delivery assistant* or birth assistant*).mp. OR ((location or place) adj3 (birth or delivery)).mp. **AND** ((geographic or physical) adj5 (location* or access* or proximit* or distance*)).mp. OR ((time or distance*) adj5 (walk* or driv* or travel*)).mp. OR (Health Services Accessibility or Spatial access*).mp. OR Access to care.mp. **AND** Observational Study.mp. OR Cohort Studies.mp. OR Case-Control Studies.mp. OR Cross-Sectional Studies.mp. OR Ecological stud*.mp. or Randomi*ed trial.mp or Clinical Trial/ or Randomized controlled trial.mp **AND** ((low or middle) adj3 (countr* or nation* or econom*)).mp. OR (Afghanistan or Benin or Burkina Faso or Burundi or Central African Republic or Chad or Comoros or Congo Democratic Republic or Eritrea or Ethiopia or Gambia or Guinea or Guinea-Bissau or Haiti or Korea Democratic People's Republic or Liberia or Madagascar or Malawi or Mali or Mozambique or Nepal or Niger or Rwanda or Senegal or Sierra Leone or Somalia or South Sudan or Tanzania or Togo or Uganda or Zimbabwe or Armenia or Bangladesh or Bhutan or Bolivia or Cabo Verde or Cambodia or Cameroon or Congo Republic or Ivory Coast or Djibouti or Egypt or El Salvador or Ghana or Guatemala or Honduras or India or Indonesia or Kenya or Kiribati or Kosovo or Kyrgyz Republic or Laos or Lesotho or Mauritania or Micronesia Federation or Moldova or Mongolia or Morocco or Myanmar or Nicaragua or Nigeria or Pakistan or Papua New Guinea or Philippines or Samoa or Sao Tome) and Principe) or Solomon Islands or Sri Lanka or Sudan or Swaziland or Syrian Arab Republic or Tajikistan or Timor-Leste or Tonga or Tunisia or Ukraine or Uzbekistan or Vanuatu or Vietnam or West Bank) and Gaza) or Yemen Republic or Zambia or Albania or Algeria or American Samoa or Angola or Argentina or Azerbaijan or Belarus or Belize or Bosnia) and Herzegovina) or Botswana or Brazil or Bulgaria or China or Colombia or Costa Rica or Cuba or Dominica or Dominican Republic or Equatorial Guinea or Ecuador or Fiji or Gabon or Georgia or Grenada or Guyana or Iran or Iraq or Jamaica or Jordan or Kazakhstan or Lebanon or Libya or Macedonia or Malaysia or Malaysia or Maldives or Marshall Islands or Mauritius or Mexico or Montenegro or Namibia or Palau or Panama or Paraguay or Peru or Romania or Russian Federation or Serbia or South Africa or Saint Lucia or Saint Vincent) and the Grenadines) or Suriname or Thailand or Turkey or Turkmenistan or Tuvalu or Venezuela).mp |
| **CINHAL** | Midwifery" OR "Maternal Health Services" OR “Perinatal Care” OR “Delivery, Obstetric” OR "Obstetric Emergencies" OR "Labor Complications" OR "Labor" OR "Labor Support" OR "Cesarean Section, Elective" OR "Cesarean Section" OR "Parturition"  OR "Childbirth" OR "Obstetric Care" OR "Delivery Care (Saba CCC)" OR "Delivery assistant*"  OR "Birth Place" OR "Birth assistant*"  OR "“(location or place) adj3 (birth or delivery)”"  OR "“(location or place) adj3 (birth or delivery)”"  **AND** "geographic distance" OR "physical distance" OR "Geographic Locations") OR "geographic proximit*" OR "physical proximit*" OR "physical location*" OR "geographic acess" OR "geographic acess" OR "physical access" OR "“(time or distance*) adj5 (walk* or driv* or travel*)”" OR "“(time or distance*) adj5 (walk* or driv* or travel*)”" OR "walk* time" OR "driv* time" OR "travel* time" OR "walk* distance" OR "driv* distance" OR "travel* distance" OR Health Services Accessibility") OR (MH "Health Services Needs and Demand" "“Access to care”" OR "“Spatial access*”" OR "Catchment Area (Health)" **AND**  "Nonexperimental Studies" OR "“Observational Study”" OR "Cross Sectional Studies") OR "“Cohort Studies”" OR "Case Control Studies" OR "“Ecological stud*”" or “Randomi*ed trial” or “Clinical Trial” or “Randomized controlled trial” **AND** "Low and Middle Income Countries") OR "Afghanistan or Benin or “Burkina Faso” or Burundi or “Central African Republic” or Chad or Comoros or “Congo Democratic Republic” or Eritrea or Ethiopia or Gambia or Guinea or Guinea-Bissau or Haiti or “Korea Democratic People's Republic” or Liberia or Madagascar or Malawi or Mali or Mozambique or Nepal or Niger or Rwanda or Senegal or “Sierra Leone” or Somalia or “South Sudan” or Tanzania or Togo or Uganda or Zimbabwe or Armenia or Bangladesh or Bhutan or Bolivia or “Cabo Verde” or Cambodia or Cameroon or “Congo Republic” or “Ivory Coast” or Djibouti or Egypt or El-Salvador or Ghana or Guatemala or Honduras or India or Indonesia or Kenya or Kiribati or Kosovo or “Kyrgyz Republic” or Laos or Lesotho or Mauritania or “Micronesia Federation” or Moldova or Mongolia or Morocco or Myanmar or Nicaragua or Nigeria or Pakistan or “Papua New Guinea” or Philippines or Samoa or “Sao Tome and Principe” or “Solomon Islands” or Sri-Lanka or Sudan or Swaziland or “Syrian Arab Republic” or Tajikistan or Timor-Leste or Tonga or Tunisia or Ukraine or Uzbekistan or Vanuatu or Vietnam or “West Bank and Gaza” or “Yemen Republic” or Zambia or Albania or Algeria or “American Samoa” or Angola or Argentina or Azerbaijan or Belarus or Belize or Bosnia and Herzegovina or Botswana or Brazil or Bulgaria or China or Colombia or “Costa Rica” or Cuba or Dominica or “Dominican Republic” or “Equatorial Guinea” or Ecuador or Fiji or Gabon or Georgia or Grenada or Guyana or Iran or Iraq or Jamaica or Jordan or Kazakhstan or Lebanon or Libya or Macedonia or Malaysia or Malaysia or Maldives or “Marshall Islands” or Mauritius or Mexico or Montenegro or Namibia or Palau or Panama or Paraguay or Peru or Romania or “Russian Federation” or Serbia or “South Africa” or “Saint Lucia” or “Saint Vincent and the Grenadines” or Suriname or Thailand or Turkey or Turkmenistan or Tuvalu or Venezuela" |
| **Scopus** | midwifery  OR  "Maternal Health Services"  OR  "Perinatal Care"  OR  delivery,  AND obstetric  OR  "Obstetric Labor Complications"  OR  labor  OR  "Caesarean Section" )  OR obstetrics  OR  parturition  OR  childbirth  OR  "Obstetric care"  OR  "Delivery care"  OR  "Delivery assistant*"  OR  "Birth assistant*"  OR  "(location or place) adj3 (birth or delivery)" )  **AND** "(geographic or physical) adj5 (location* or access* or proximit* or distance*)"  OR  "(time or distance*) adj5 (walk* or driv* or travel*)" )  OR  "Health Services Accessibility"  OR  "Spatial access*"  OR  "Catchment Area (Health)"  OR  "Access to care" )  **AND** Observational Study"  OR  "Cohort Studies"  OR  "Case-Control Studies"  OR  "Cross-Sectional Studies"  OR  "Ecological stud*" )  or “Randomi*ed trial” or “Clinical Trial” or “Randomized controlled trial” **AND** "(low or middle) adj3 (countr* or nation* or econom*)" OR Afghanistan OR Benin or “Burkina Faso” OR Burundi OR “Central African Republic” OR Chad OR Comoros OR “Congo Democratic Republic” OR Eritrea or Ethiopia OR Gambia OR Guinea OR Guinea-Bissau OR Haiti OR “Korea Democratic People's Republic” OR Liberia OR Madagascar OR Malawi OR Mali OR Mozambique OR Nepal OR Niger OR Rwanda OR **Senegal** OR **“**Sierra Leone” OR Somalia OR “South Sudan” OR Tanzania OR Togo OR Uganda OR Zimbabwe OR Armenia OR Bangladesh OR Bhutan OR Bolivia OR “Cabo Verde” OR **Cambodia** OR Cameroon OR “Congo Republic” OR “Ivory Coast” OR Djibouti OR Egypt OR El-Salvador OR Ghana OR Guatemala OR Honduras OR India OR Indonesia OR Kenya OR Kiribati OR Kosovo OR “Kyrgyz Republic” OR Laos OR Lesotho OR Mauritania OR “Micronesia Federation” OR Moldova OR **Mongolia** OR Morocco OR Myanmar OR Nicaragua OR Nigeria OR Pakistan OR **“**Papua New Guinea” OR Philippines OR Samoa OR *“*Sao Tome and Principe” OR “Solomon Islands” OR Sri-Lanka OR Sudan OR Swaziland OR “Syrian Arab Republic” OR Tajikistan OR Timor-Leste OR **Tonga** OR **Tunisia** OR Ukraine OR Uzbekistan OR Vanuatu OR Vietnam OR “West Bank and Gaza” OR “Yemen Republic” OR Zambia OR Albania OR Algeria OR “American Samoa” OR Angola OR **Argentina** OR Azerbaijan OR Belarus OR Belize OR Bosnia and Herzegovina OR Botswana OR Brazil OR Bulgaria OR China OR Colombia OR “Costa Rica” OR Cuba OR Dominica OR “Dominican Republic” OR “**Equatorial Guinea”** OR Ecuador OR Fiji OR Gabon OR **Georgia** OR Grenada OR **Guyana** OR Iran OR Iraq OR Jamaica OR Jordan OR Kazakhstan OR Lebanon OR Libya OR Macedonia OR Malaysia OR Malaysia OR Maldives OR “Marshall Islands” OR Mauritius OR Mexico OR Montenegro OR Namibia OR Palau OR Panama OR Paraguay OR Peru OR Romania OR “**Russian Federation”** OR Serbia OR “South Africa” OR “Saint Lucia” OR “Saint Vincent and the Grenadines” OR Suriname OR Thailand OR Turkey OR Turkmenistan OR Tuvalu OR **Venezuela** |
| **Hand search: Google Scholar** | Varieties of key terms from the above-mentioned were used; and 16 papers turned out. |
