# Supplementary material for: The impact of geographic access on institutional delivery care use in low and middle-income countries: Systematic review and meta-analysis
Source: PLoS One. 2018 Aug 30;13(8):e0203130. doi: 10.1371/journal.pone.0203130 (PMC6117044; doi:10.1371/journal.pone.0203130)
Supplement: S2 Table — (DOCX) [file pone.0203130.s002.docx]

| Citation | Free of selection bias? | Appropriate sampling? | Adequate sample size? | Detail sample description? | Free of coverage bias? | Free of measurement/ classification bias? | Measurement reliability? | Appropriate statistical analysis? | Adequate response rate? | Overall |
| --- | --- | --- | --- | --- | --- | --- | --- | --- | --- | --- |
| Kawakatsu et al, 2014 | **Yes** | **Yes** | **Yes** | **Yes** | **Yes** | **Yes** | **Yes** | **Yes** | **Yes** | **9/9** |
| Hailu & Berhe, 2014 | **Yes** | **Yes** | **Yes** | **Yes** | **Yes** | **Unclear** | **Yes** | **Yes** | **Yes** | **8/9** |
| Habte & Demissie, 2015 | **Yes** | **No** | **Yes** | **Yes** | **Yes** | **Unclear** | **Yes** | **Yes** | **Yes** | **7/9** |
| Joshi et al, 2016 | **No** | **Yes** | **No** | **Yes** | **No** | **Yes** | **Yes** | **No** | **No** | **3/9** |
| Wagle et al, 2004 | **Yes** | **Yes** | **Yes** | **Yes** | **Unclear** | **Yes** | **Yes** | **Unclear** | **Yes** | **7/9** |
| Jain et al, 2015 | **Yes** | **Yes** | **Yes** | **Yes** | **Yes** | **Yes** | **Yes** | **Yes** | **Yes** | **9/9** |
| De Allegri et al, 2011 | **No** | **No** | **Unclear** | **Yes** | **Yes** | **Yes** | **Unclear** | **Yes** | **Unclear** | **4/9** |
| Lohela et al, 2012 | **Yes** | **Yes** | **Yes** | **Yes** | **Yes** | **Yes** | **Yes** | **Yes** | **Not applicable** | **9/9** |
| Gabrysch et al, 2011 | **Yes** | **Yes** | **Yes** | **Yes** | **Yes** | **Yes** | **Yes** | **Yes** | **Not applicable** | **9/9** |
| Anyait et al, 2012 | **No** | **No** | **Yes** | **Yes** | **Yes** | **Yes** | **Yes** | **Yes** | **Yes** | **7/9** |
| Joharifard et al, 2012 | **No** | **No** | **Unclear** | **Yes** | **Unclear** | **Yes** | **Yes** | **Yes** | **Yes** | **5/9** |
| Zegeye et al, 2014 | **No** | **Yes** | **Yes** | **Yes** | **Yes** | **Yes** | **Yes** | **Unclear** | **Yes** | **7/9** |
| Masters et al, 2013 | **Yes** | **Yes** | **Yes** | **Yes** | **Yes** | **Yes** | **Yes** | **Yes** | **Not applicable** | **9/9** |
| De Allegri et al, 2015 | **Unclear** | **Unclear** | **Unclear** | **Yes** | **Yes** | **Yes** | **Yes** | **Yes** | **Not applicable** | **6/9** |
| Worku & Alemay, 2016 | **Yes** | **No** | **Yes** | **No** | **Yes** | **Yes** | **Yes** | **No** | **Yes** | **6/9** |
| Van et al, 2006 | **Yes** | **No** | **Yes** | **No** | **No** | **Yes** | **Yes** | **Unclear** | **Yes** | **5/9** |
| Lwelamira et al, 2012 | **Yes** | **Unclear** | **Unclear** | **Yes** | **Yes** | **Yes** | **Yes** | **Yes** | **Yes** | **7/9** |
| Yanagisawa et al, 2006 | **Yes** | **Yes** | **Unclear** | **Yes** | **Yes** | **Yes** | **Yes** | **Yes** | **Yes** | **8/9** |
| Gage & Guirle, 2006 | **Yes** | **Yes** | **Yes** | **Yes** | **Yes** | **Yes** | **Yes** | **Yes** | **Yes** | **9/9** |
| Kesterton et al, 2012 | **Yes** | **Yes** | **Yes** | **Yes** | **Yes** | **Yes** | **Yes** | **Yes** | **Yes** | **9/9** |
| Mageda & Mmbaga, 2015 | **Yes** | **Yes** | **Yes** | **Yes** | **Yes** | **Yes** | **Yes** | **Unclear** | **Yes** | **8/9** |
| Faye et al, 2011 | **Unclear** | **Unclear** | **Yes** | **No** | **Yes** | **Yes** | **Yes** | **Yes** | **Yes** | **6/9** |
| Kitui et a, 2013 | **Yes** | **Yes** | **Yes** | **Yes** | **Yes** | **Yes** | **Yes** | **Yes** | **Yes** | **9/9** |
| Ogolla, 2015 | **No** | **No** | **Unclear** | **No** | **Yes** | **Yes** | **Yes** | **Yes** | **Yes** | **9/9** |
| Kumar et al, 2014 | **Yes** | **Yes** | **Yes** | **Yes** | **Yes** | **Yes** | **Yes** | **Yes** | **Yes** | **9/9** |
| Hounton et al, 2008 | **Yes** | **Yes** | **Yes** | **Yes** | **Yes** | **Yes** | **Yes** | **Yes** | **Yes** | **9/9** |
| Teferra et al, 2012 | **Yes** | **Yes** | **Yes** | **Yes** | **Yes** | **Yes** | **Yes** | **Yes** | **Yes** | **9/9** |
| Amano et al, 2012 | **Yes** | **Yes** | **Yes** | **No** | **Yes** | **Yes** | **Yes** | **Yes** | **Yes** | **8/9** |
| Shimazaki et al, 2013 | **Yes** | **Unclear** | **Unclear** | **No** | **Yes** | **Yes** | **Yes** | **Yes** | **Yes** | **6/9** |
