# Supplementary material for: The impact of geographic access on institutional delivery care use in low and middle-income countries: Systematic review and meta-analysis
Source: PLoS One. 2018 Aug 30;13(8):e0203130. doi: 10.1371/journal.pone.0203130 (PMC6117044; doi:10.1371/journal.pone.0203130)
Supplement: S3 Table — (DOCX) [file pone.0203130.s003.docx]

| Criteria | Free of selection bias? | Clear exposure measurement? | Valid & reliable exposure measurement? | Identified confounders? | Confounder management strategies stated? | Free of outcome sample? | Valid & reliable outcome measure? | Sufficient follow-up time? | Follow-up complete? | Incomplete follow-up strategies? | Appropriate statistical analysis? | Overall |
| --- | --- | --- | --- | --- | --- | --- | --- | --- | --- | --- | --- | --- |
| Karkee et al, 2013 | **Unclear** | **Unclear** | **Unclear** | **Yes** | **Yes** | **Yes** | **Yes** | **Unclear** | **Unclear** | **Unclear** | **No** | **4/11** |
