# Supplementary material for: The impact of geographic access on institutional delivery care use in low and middle-income countries: Systematic review and meta-analysis
Source: PLoS One. 2018 Aug 30;13(8):e0203130. doi: 10.1371/journal.pone.0203130 (PMC6117044; doi:10.1371/journal.pone.0203130)
Supplement: S4 Table — (DOCX) [file pone.0203130.s004.docx]

| Criteria | Free of selection bias? | Cases & controls matched? | Same identification criteria for cases & controls | Valid & reliable exposure measurement? | Same exposure measure for cases & controls | Identified confounders? | Confounder management strategies stated? | Valid & reliable outcome measure? | Sufficient exposure time? | Appropriate statistical analysis? | Overall |
| --- | --- | --- | --- | --- | --- | --- | --- | --- | --- | --- | --- |
| Feyissa & Genemo, 2014 | Yes | Yes | Yes | Yes | Yes | No | No | Yes | Not applicable | No | 7/10 |
